# Design of Protein Multi-specificity Using an Independent Sequence Search Reduces the Barrier to Low Energy Sequences

**DOI:** 10.1371/journal.pcbi.1004300

**Published:** 2015-07-06

**Authors:** Alexander M. Sevy, Tim M. Jacobs, James E. Crowe, Jens Meiler

**Affiliations:** 1 Center for Structural Biology, Vanderbilt University, Nashville, Tennessee, United States of America; 2 Department of Biochemistry, University of North Carolina at Chapel Hill, Chapel Hill, North Carolina, United States of America; 3 Vanderbilt Vaccine Center, Vanderbilt University Medical Center, Nashville, Tennessee, United States of America; 4 Department of Chemistry, Vanderbilt University, Nashville, Tennessee, United States of America; La Jolla Institute for Allergy and Immunology, UNITED STATES

## Abstract

Computational protein design has found great success in engineering proteins for thermodynamic stability, binding specificity, or enzymatic activity in a ‘single state’ design (SSD) paradigm. Multi-specificity design (MSD), on the other hand, involves considering the stability of multiple protein states simultaneously. We have developed a novel MSD algorithm, which we refer to as REstrained CONvergence in multi-specificity design (RECON). The algorithm allows each state to adopt its own sequence throughout the design process rather than enforcing a single sequence on all states. Convergence to a single sequence is encouraged through an incrementally increasing convergence restraint for corresponding positions. Compared to MSD algorithms that enforce (constrain) an identical sequence on all states the energy landscape is simplified, which accelerates the search drastically. As a result, RECON can readily be used in simulations with a flexible protein backbone. We have benchmarked RECON on two design tasks. First, we designed antibodies derived from a common germline gene against their diverse targets to assess recovery of the germline, polyspecific sequence. Second, we design “promiscuous”, polyspecific proteins against all binding partners and measure recovery of the native sequence. We show that RECON is able to efficiently recover native-like, biologically relevant sequences in this diverse set of protein complexes.

## Author Summary

The ability to design a new protein with a desired activity has been a longstanding goal of computational biologists, to create proteins with new binding activity or increased stability. An even more ambitious goal is multi-specificity design, which extends general protein design by creating a sequence that has low energy with multiple binding partners. We have developed a new algorithm for multi-specificity design that more efficiently finds a low energy sequence for all complexes. This increased efficiency enables simulation of biologically relevant motion between binding partners, such as backbone movement and shifts in orientation. We show that our algorithm outperforms existing approaches, and compare the predicted low energy sequences to the sequences naturally seen through evolution of each protein. We find that this algorithm is able to more accurately represent the scope of sequences that are found in biological contexts. This method can be applied to design new proteins with the ability to bind multiple distinct partners.

## Introduction

Computational protein design is an invaluable tool for protein engineers seeking to create a protein with novel properties. Protein design, also known as the inverse folding problem, involves searching for a sequence that stabilizes a given conformation. Besides the obvious goal–to give the protein increased thermodynamic stability [1–3]–protein design often pursues the goal of creating new function. This can include for example redesigning an antibody to recognize a new variant of a target protein [4], designing an enzyme to bind the transition state for a new chemical reaction [5], or redesigning a DNA-binding protein to recognize a different DNA sequence [6]. Most success in protein design has been achieved through a single state design (SSD) task, i.e. the free energy minimization of a single protein conformation to increase its stability [1,7,8].

### Multistate design approaches

In contrast to SSD, multistate design (MSD) minimizes the free energy of multiple protein conformations (“states”) simultaneously. This enables negative design, which involves destabilizing a certain conformation to shift relative occupancy to alternate conformations, which is useful in designing proteins with binding selectivity. MSD has been applied successfully in a number of cases, including the design of a protein conformational switch [9], design of selective b-ZIP binding peptides [10], and design of an enzyme with DNA cleavage specificity [11], among others [12,13].

### Algorithmic requirements for multistate design

All MSD algorithms have at their core a fitness function that defines the favorability of a given sequence based on its corresponding energy in each state. The major challenge in fixed backbone MSD is efficient optimization of side chain rotational isomer (“rotamer”) placement, using the fitness function as the objective function. As more states are considered it becomes increasingly difficult to find the minimum energy sequence on a fixed backbone. As the same sequence on all states is constrained, extensive sampling in sequence and rotamer space is required. This is often accomplished via thorough but slow genetic algorithms [12,14,15].

### Challenges in expanding the scope of multistate design

This difficulty in reaching the global minimum in a basic fixed backbone design problem precludes the possibility of using alternate sampling strategies, such as iterating between backbone minimization and rotamer optimization. However, these techniques have been used in SSD to great effect and are often critical to find the lowest energy conformation and sequence [1,8]. In result, MSD algorithms can arrive at an incorrect solution even after successful sequence optimization just because the fixed backbone precludes the lowest energy sequence and conformation from being sampled. This issue can be partially resolved by the inclusion of multiple backbone conformations as separate states [16]. However, there is a need for a method that can more efficiently reach the optimal MSD solution for an arbitrary number of input states without relying on the commonly held “fixed backbone assumption”.

### Multi-specificity design as single state design with restraints

To this end, we have developed a novel MSD algorithm, referred to as REstrained CONvergence in multi-specificity design (RECON). The algorithm is based on a different conception of MSD, wherein each state independently explores sequence space to reach its energetic minimum. A step-wise increasing convergence restraint is applied such that corresponding positions in different states converge on the same amino acid. By encouraging sequence convergence between different states rather than enforcing a single sequence, we hypothesize that energetic barriers to the fittest solution collapse, reducing the ruggedness of the energetic landscape in a MSD problem to SSD-like complexity. In result the search efficiency and speed are substantially increased allowing for the sampling of additional degrees of freedom. Further, we hypothesize that including backbone conformational sampling reduces the chance that the low energy and possibly correct solutions are excluded from the search space.

## Results

### The restrained convergence algorithm

**Fig 1:**
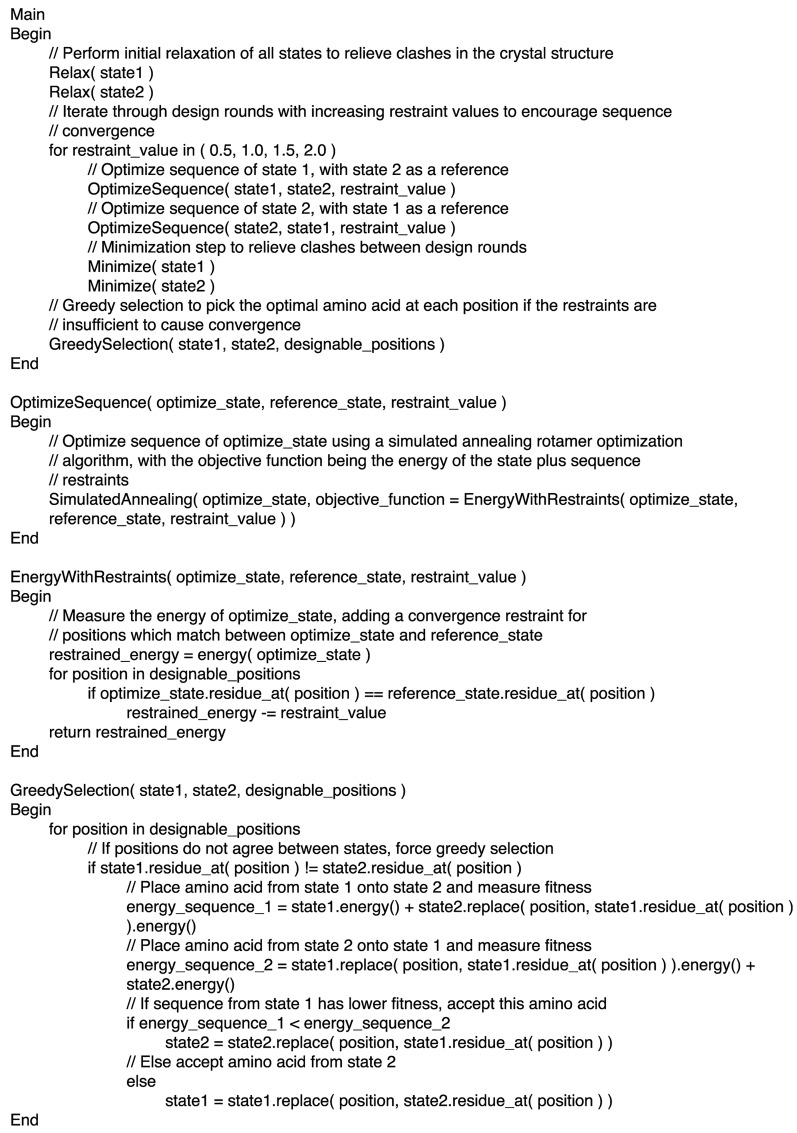
Pseudocode describing the implementation of the RECON algorithm.

The RECON algorithm allows separate states to explore their own local sequence and conformational space to optimize free energy, while restraining corresponding residues in different states with a convergence restraint to encourage sequence convergence. Convergence restraints are kept small in early rounds, to allow each state to explore its own lowest energy sequence, and ramped up in later rounds to encourage sequence convergence between different states. This is followed by a greedy selection step, which evaluates all candidate amino acids at positions that fail to converge, and selects the one that results in the lowest fitness when applied over all states. This greedy selection is included in order to ensure that one multi-specific sequence is generated from each design trajectory. Backbone minimization steps can be included between design rounds to relieve slight clashes between side chains. Pseudocode describing the implementation of the algorithm is shown in Fig 1. Individual states optimize rotamer placement using a simulated annealing Monte Carlo search, sampling from a predefined rotamer library [17,18]. However, we emphasize that this method can be applied to any multi-specificity problem using an arbitrary optimization method and scoring function.

### Reduction of energy barriers in restrained multi-specificity design

**Fig 2:**
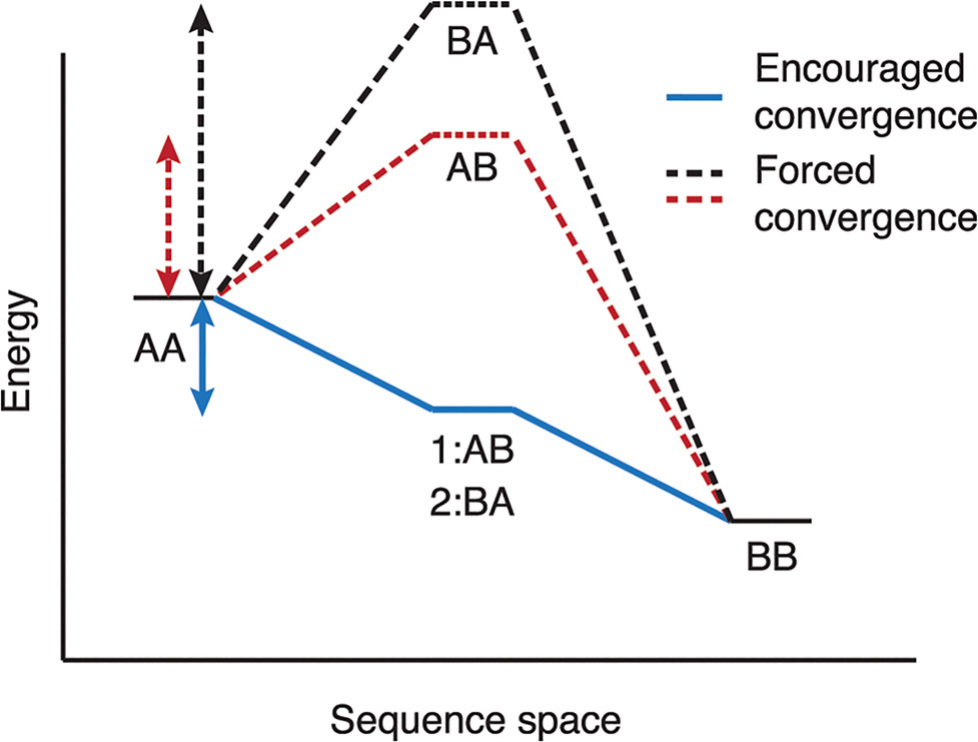
Schematic showing proposed energy landscape of forced vs. encouraged sequence convergence in MSD. By allowing each state to maintain its own sequence and explore sequence space independently, RECON is able to provide an intermediate solution in a MSD problem, enabling more rapid determination of a low energy solution. Dashed lines represent forced convergence, where both states must adopt the same sequence (either AB or BA), whereas the solid line represents encouraged convergence, where state 1 can adopt sequence AB while state 2 adopts BA. This creates a lower energy intermediate state leading to more rapid adoption of the optimal solution, sequence BB.

By allowing each state to determine its optimal sequence independently, we can collapse the energy barrier to reaching a “compromised” sequence that results in low energy in all states. We propose a scenario in which encouraging sequencing convergence in this way can reduce the energetic barrier and enable convergence on a low energy solution (Fig 2). In this scenario, two separate mutations from residue identity A to B are needed for the lowest fitness over both states. Each single mutation will encounter a high energy penalty and rarely selected by a genetic algorithm–only when both mutations are stochastically placed together will the solution emerge, which may take a large number of evaluations. However, when sequence convergence is encouraged rather than enforced, each state will identify an intermediate solution in early rounds, and in later rounds the most favorable solution will be selected from the differing states. This collapses the barrier on the pathway to a favorable solution and reduces the steps necessary to find that solution.

### Common germline gene reversion benchmark

To benchmark RECON, we considered two types of design problems. In the first, mature antibodies derived from a common germline gene were entered into MSD in complex with their target antigens. It has been shown that MSD of mature antibodies results in a higher rate of germline sequence reversion than SSD, implying that the germline sequence is near-optimal for polyspecificity [19,20]. Therefore, we designed each antibody against its respective targets and used germline sequence recovery as an indirect measure of the rate of recovery of an optimal solution. We used antibodies derived from three different germline genes—V_H_1-69, V_H_3-23, and V_H_5-51. The number of antibody-antigen complexes per germline gene ranged from 3 to 6 (Table 1).

**Table 1:** Complexes used in common germline antibody benchmark.

V_H_ germline gene	Variable positions^a^	Antibody	Ligand	PDB ID
V_H_1-69	40	D5	gp41	2CMR
		F10	H5/Vietnam/1203/2004	3FKU
		CR6261	H5/Vietnam/1203/2004	3GBM
		8066	gp41	3MA9
		8062	gp120	3MAC
		1281	gp41	3P30
V_H_3-23	31	Pertuzumab	ErbB2	1S78
		G6	VEGF	2FJG
		Apu2.16	Ubiquitin	3DVN
		E2	MT-SP1	3BN9
V_H_5-51	30	2219	UG1033	2B1A
		K1-70	TSHR	2XWT
		Ustekinumab	IL-12	3HMX

RECON was benchmarked on three sets of mature antibodies derived from the same V_H_ gene. Effective MSD should result in reversion of mature antibodies to the polyspecific germline sequence.

^a^Germline sequence and positions varying from germline are inferred from IMGT/3D Structure-DB [21].

### Promiscuous protein design benchmark

The second task was to design a set of “promiscuous” proteins, proteins that have been crystallized in complex with multiple binding partners, against each of these partners. Similar to polyspecific germline antibodies, promiscuous proteins have been shown to have a native sequence that is near-optimal for binding to all of the partners [14,22]. Therefore an effective MSD protocol would result in a high rate of native sequence recovery. A set of five promiscuous proteins derived from a study done by Humphris et al. was used [14], in addition to two broadly neutralizing anti-influenza hemagglutinin antibodies (Table 2) [23,24].

**Table 2:** Complexes used in promiscuous protein benchmark.

Promiscuous protein	Binding partner	PDB ID	Designable positions^a^
CR6261	H5/Vietnam/1203/2004	3GBM	19
	H1/BrevigMission/1/1918	3GBN	
FI6v3	H1/California/04/2009	3ZTN	21
	H3/Aichi/2/1968	3ZTJ	
CheY	FLiM	1F4V	15
	CheA	1FFG	
	CheZ	1KMI	
Elastase	Elastase Inhibitor	1EAI	25
	Elafin	1FLE	
	Hybrid Squash Inhibitor	1MCV	
FYN SH3 Domain	HIV-1 NEF Protein	1AVZ	7
	SAP	1M27	
PapD Chaperone	PapE	1N0L	28
	PapK	1PDK	
	PapD Homodimer	1QPP	
Ran	Importin beta	1IBR	24
	Exportin CSE1P/KAP60P	1WA5	

RECON was benchmarked on a set of promiscuous proteins that have been crystallized in complex with multiple partners. As the native sequence is near optimal for binding of all partners, MSD should recover the native sequence at a high rate.

^a^Residues determined to be at the interface with all binding partners. See methods for details on interface residue calculations.

### Design algorithms included in benchmark

Benchmark cases were designed using three separate design methods. First, design was performed using RECON with a fixed backbone. Fixed backbone design has to this point been the standard in MSD due to the complexity involved in recalculating rotamer interactions for each backbone movement. However, using fixed backbone design alone is prone to false negatives, as sequences that may be highly favorable with a small shift in backbone conformation are discarded. One of the unique advantages of RECON is its ability to incorporate iterative rounds of rotamer packing and backbone minimization. Therefore, we included such an iterative protocol as the second approach in our benchmark. For comparison purposes, all complexes were also designed using the existing MSD application in Rosetta (MPI_MSD), which operates on a fixed backbone [15]. MPI_MSD differs from RECON in that it uses a genetic algorithm to create and advance mutations and a user-defined fitness function to assess fitness of each sequence. However, as both methods are built into the Rosetta framework, they sample from the same rotamer library and use the same scoring function and are therefore suitable for comparison. In addition to native sequence recovery, we used the fitness of the top ten designs, defined as the sum of Rosetta energies of all complexes, to analyze how effectively each protocol reached an energetic minimum. This fitness function has been previously used in studies of design of protein multi-specificity [14]. We use the term “design” to refer to sequence optimization of existing protein-protein complexes—however, it is important to note that these sequences were not experimentally characterized, and results reported are purely *in silico*.

### Common germline derived antibodies

**Fig 3:**
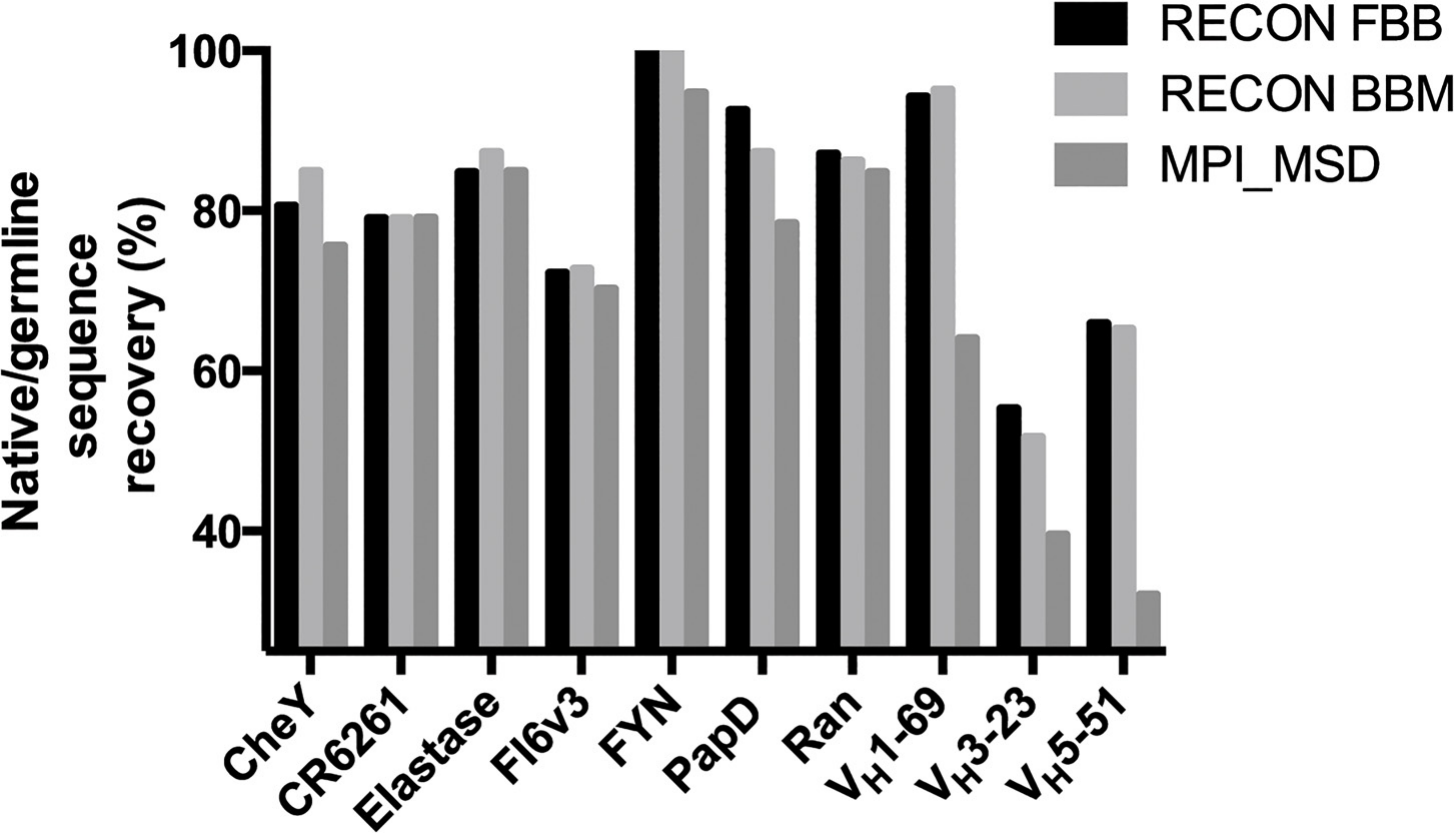
Native/germline sequence recovery of designed complexes. 100 designs were generated using RECON, with both fixed backbone (FBB) and backbone minimized (BBM) protocols, and MPI_MSD. Sequences of the top 10% of models were compared to either the native sequence or, in the case of common germline-derived antibodies, to the germline sequence. See methods for details of native sequence recovery calculations.

For common germline gene-derived antibodies, RECON was consistently able to recover the germline sequence at a higher rate than MPI_MSD (Table 3 and Fig 3). Germline sequence recovery for RECON ranged from 55–94% using fixed backbone and 51–95% using backbone minimization, while recovery for designs using MPI_MSD ranged from 32–64%. When comparing RECON fixed backbone to MPI_MSD, it appeared that designs created by RECON, although higher in native sequence content, were also energetically less favorable. We therefore subjected all fixed backbone designs to a single round of Rosetta relax energy minimization to relieve frustrations and allow for direct comparison of fitness of RECON incorporating backbone minimization to fixed backbone designs (S1 Table). These post-minimization fitness values show that the energetic gap between RECON- and MPI_MSD-generated designs was substantially closed, and that designs generated by any method occupied similar ranges of fitness. We observed that MPI_MSD tended to produce designs with the lowest fitness—however, it is important to note that rotamer optimization within Rosetta is a stochastic process, with no guarantee of reaching the global minimum. Therefore a protocol that performs hundreds of rounds of rotamer optimization, such as MPI_MSD, would be expected to produce better energies than one performing four rounds of optimization, such as RECON, independent of the sequence identity of structures being optimized.

**Table 3:** Results of common germline gene multi-specificity design benchmark.

	Native Sequence Recovery (%)			Fitness^a^ (REU)		
Germline gene	RECON FBB	RECON BBM	MPI_MSD	RECON FBB	RECON BBM	MPI_MSD
**V**_**H**_ **1-69**	94.2	95.1	64.0	-3015.9	-5306.7	-5131.9
**V**_**H**_ **3-23**	55.3	51.7	39.5	-911.7	-3427.1	-3320.9
**V**_**H**_ **5-51**	65.9	65.2	32.0	-840.5	-2348.3	-2260.8
**Average**	**71.8**	**70.7**	**45.2**	**-1589.4**	**-3694.0**	**-3571.2**

Three sets of antibodies encoded by a common germline gene were designed against their targets using RECON, both by fixed backbone (FBB) and backbone minimized (BBM) designs, and MPI_MSD algorithms. Designs were evaluated by recovery of the germline sequence and the fitness of designed models.

^a^Fitness is defined as the sum of the Rosetta score of all input complexes, in Rosetta Energy Units (REU). Fitness was reported for the top ten complexes generated by each design method.

### Promiscuous protein complexes

RECON was able to recover the native sequence at a very high level for all promiscuous protein complexes–native sequence recovery ranged from 72–100% and 73–100%, using fixed backbone and backbone minimization, respectively. MPI_MSD generated designs with native sequence recovery ranging from 70–94% (Table 4 and Fig 3). In most cases fitness of designs generated by RECON fixed backbone and MPI_MSD were very similar, suggesting that both methods have reached the energetic minimum (S1 Table). Even though all methods reached a similar level of native sequence recovery and energetic fitness in a majority of these benchmark cases, RECON was able to reach these minima by searching a compressed sequence space, allowing for increased computational efficiency.

**Table 4:** Results of promiscuous protein multi-specificity design benchmark.

	Native Sequence Recovery (%)			Fitness^a^ (REU)		
Promiscuous protein	RECON FBB	RECON BBM	MPI_MSD	RECON FBB	RECON BBM	MPI_MSD
**CheY**	80.6	84.9	75.6	-1093.1	-1119.7	-1090.8
**CR6261**	79.0	79.0	79.1	-2499.5	-2537.7	-2499.9
**Elastase**	84.8	87.3	84.9	-1383.8	-1445.1	-1394.7
**FI6v3**	72.2	72.7	70.2	-2459.1	-2515.2	-2464.3
**FYN**	100.0	100.0	94.7	-758.3	-780.3	-760.8
**PapD**	92.5	87.3	78.4	-1685.5	-1789.2	-1758.6
**Ran**	87.1	86.2	84.8	-2682.3	-3716.4	-3696.5
**Average**	**85.2**	**85.3**	**81.1**	**-1794.5**	**-1986.2**	**-1952.2**

Designs were generated using RECON, both by fixed backbone (FBB) and backbone minimized (BBM) designs, and MPI_MSD algorithms. Models were evaluated by recovery of the native starting sequence, and the energetic fitness of design models.

^a^Fitness is defined as the sum of the Rosetta score of all input complexes, in Rosetta Energy Units (REU). Fitness was reported for the top ten complexes generated by each design method.

### Importance of ramping convergence restraints on algorithm performance

We hypothesized that gradually ramping the convergence restraints will allow for sequence divergence in early rounds of design and enforce convergence in later rounds, leading to an improved result as it smoothens the energy landscape. To confirm the effects of gradually increasing the weight of the convergence restraint, we performed a control in which sequences were designed independently for each state with no convergence restraint, followed by sequence selection by the greedy selection used at the end of RECON (S2 Table). This greedy selection algorithm performed significantly worse than RECON with gradual ramping convergence restraints, with worse native sequence recovery in all benchmark cases but one. In addition, in many benchmark cases fitness was significantly worsened for designs generated by this greedy selection protocol. These results indicate that ramping convergence restraints throughout the design protocol is critical for the increased performance of RECON.

### Sequence recovery at positions that fail to converge

Based on the decreased performance of this greedy selection algorithm, it would be expected that RECON works best at positions where amino acids converge between different states by the end of the protocol and are not greedily selected. We therefore evaluated the convergence of amino acids at each position for the V_H_5-51 benchmark set. We report the number of times a position failed to converge in 100 design trajectories for the 30 designed positions in this benchmark set (S3 Table). The results suggest that most positions tend to be consistent in their patterns of convergence, and that the majority (21 out of 30) reach a common amino acid solution by the end of the protocol. The results of the greedy selection protocol suggest that failure to converge leads to a decrease in performance of the algorithm and selection of non-native amino acids. We therefore compared germline sequence recovery for positions that failed to converge in at least half of the design trajectories, as compared to those that converged in more than half of the trajectories, to determine whether these positions are substantially decreasing overall germline sequence recovery (S3 Table). Surprisingly, positions that failed to converge actually showed a higher rate of germline sequence recovery than those that were able to converge through the application of convergence restraints (S3 Table). These results indicate that, although the greedy selection algorithm should not be applied without first ramping convergence restraints to encourage convergence, the use of greedy selection for positions that fail to converge is not a limiting factor for obtaining high native sequence recovery.

### RECON is able to circumvent high-energy intermediates

**Fig 4:**
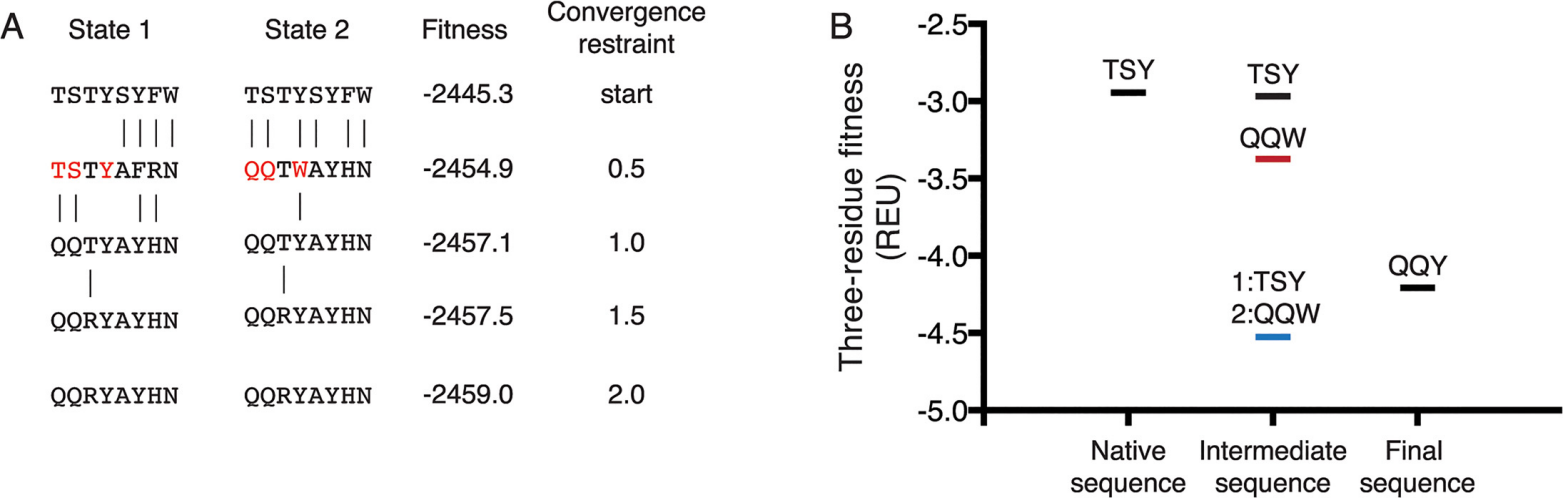
Encouraging sequence convergence in RECON can avoid high-energy sequence intermediates. A. An example design trajectory of RECON in the FI6v3 benchmark through four design rounds is shown. Sequences tend to diverge in early rounds when convergence restraints are kept low, whereas in later rounds when restraints are increased states are encouraged to adopt a single solution. The figure displays one example from the fixed backbone design protocol, with convergence restraints removed before reporting fitness. The two states showed different preferences for residues highlighted in red. B. Residues highlighted in panel A were applied to the opposing state to analyze the energetic barrier of forced sequence convergence. The energy of these three residues was analyzed when the sequence favored by state 1 (TSY) was applied to state 2, and vice versa with the sequence QQW (intermediate sequence, black/red lines). This was compared to the three-residue fitness when each state was allowed to adopt its own preferred sequence (intermediate sequence, blue line). Energies were compared to the final, “compromised” sequence (QQY). These three amino acids occurred at positions 28, 30, and 53, respectively.

In the scenario proposed in Fig 2, we hypothesize that RECON is able to circumvent high-energy intermediate sequences by encouraging rather than enforcing sequence convergence. We therefore analyzed the sequence trajectory of an example from the FI6v3 benchmark to support this scenario (Fig 4A). In early rounds, the two states diverge in sequence to explore their own energy landscapes. As restraints are increased in later rounds the two states converge on a compromised sequence that is the multi-specific solution for both, only adopting mutations when they are beneficial to both states. Although fitness values continue to decrease after encountering the compromised sequence, this is primarily due to the stochastic nature of rotamer optimization, such that increased optimization will result in a lower score. We focused on a set of complementary mutations that diverged in early rounds with a low convergence restraint, to test the hypothesis that the sequence preference of one state results in a high energy on the other state, and vice versa (Fig 4A, highlighted in red). We found that the sequences preferred by state 1 (TSY) and state 2 (QQW) indeed resulted in higher energy when forcing one state to adopt both sequences than when each state was allowed to adopt its own sequence (Fig 4B). This lowers the barrier to reaching the “compromised” sequence, adopting residues favorable to both state 1 and state 2, which in this case is the sequence QQY. Although this barrier is not as large as proposed in Fig 2, we expect that this barrier will be lower in cases where two binding partners have highly similar binding surfaces, as is the case in our benchmark sets. However, when binding surfaces are more dissimilar, and therefore finding compromise residues is more critical to a favorable binding energy, we expect this barrier to be larger, and the benefit of an independent sequence search to be even greater.

### Computational efficiency of design methods

In addition to measuring the sequence recovery and energetic fitness, we compared the computational efficiency of these three design protocols. We argue that, although in certain cases all methods were able to reach the same energetic minimum, RECON provides an added benefit in that it reached this minimum in a fraction of the time required to run MPI_MSD. To this end we compared CPU hours of runtime for generating the previously discussed designs (Table 5). As expected, RECON using a fixed backbone was the most efficient of the three protocols, followed by RECON incorporating backbone minimization, and MPI_MSD. This increase in efficiency is due to the reduction in search space by allowing each state to adopt its own sequence.

**Table 5:** Comparison of CPU runtimes for multi-specificity design using different algorithms.

	CPU Hours		
Benchmarkcase	RECON FBB	RECON BBM	MPI_MSD
**CheY**	12.0	24.0	61.1
**CR6261**	20.8	66.0	137.5
**Elastase**	24.2	47.7	198.9
**FI6v3**	21.2	80.2	46.1
**FYN**	0.8	12.8	21.2
**PAPD**	37.9	99.5	129.1
**Ran**	23.1	153.3	276.9
**V**_**H**_ **1-69**	48.7	171.1	487.1
**V**_**H**_ **3-23**	43.7	98.1	167.5
**V**_**H**_ **5-51**	19.5	71.7	95.7
**Average**	**25.2**	**82.4**	**162.1**

Runtimes in CPU hours for generation of 100 designs using RECON, both by fixed backbone (FBB) and backbone minimized (BBM) methods, and MPI_MSD algorithms.

### Generation of evolutionary sequence profiles

**Fig 5:**
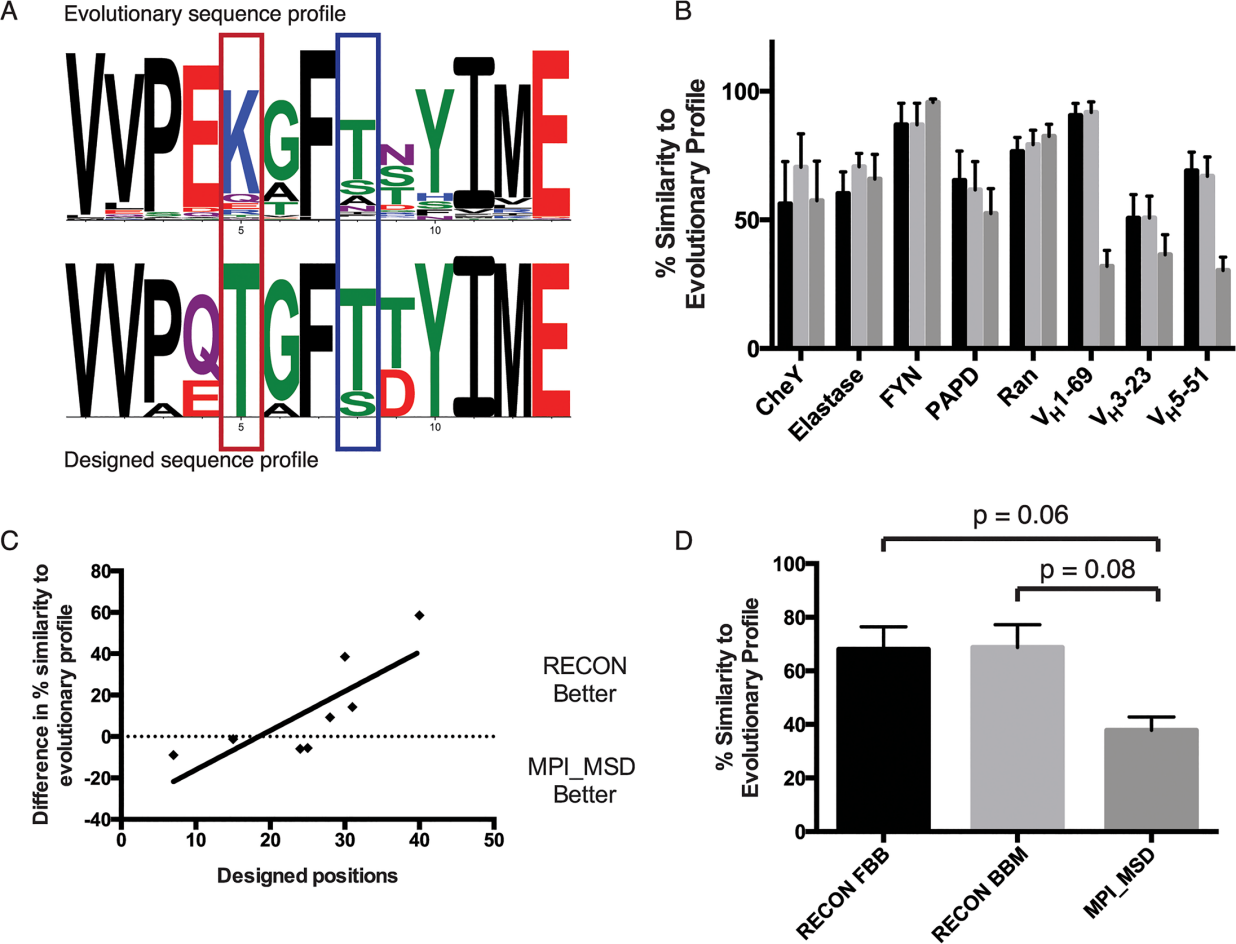
Recapitulation of evolutionary sequence profiles by multi-specificity design. A. For each protein in the benchmark set, an evolutionary sequence profile (top) was calculated and compared to the sequences generated by MSD (bottom). A similarity score was calculated for each position and averaged over designed positions to measure how well design searches biologically relevant sequence space. Highlighted are example positions where designed sequences either agreed (blue) or disagreed (red) with naturally occurring sequences. The figure displays the designed amino acid profile for a subset of positions in the V_H_5-51 benchmark set. See methods for details on percent similarity calculation. Amino acids are colored according to chemical properties. B. RECON-generated designs were more similar to observed evolutionary sequence profiles than those produced by MPI_MSD. Percent similarity was averaged over designed positions that had been mutated by any design method. Plotted are mean and SEM values. Design protocols are colored as in panel D. C. Improvement in recapitulating evolutionary sequence profiles of RECON increases with the number of designed positions. For each benchmark set, the number of designed positions is plotted against the difference in evolutionary sequence similarity between RECON backbone minimized and MPI_MSD. Least-squares linear fit is shown, with an R-value of 0.61 and p value of 0.02. D. Difference in recapitulation of evolutionary sequence profile for the four largest benchmark sets by designs generated by RECON using fixed backbone (FBB) or backbone minimization (BBM) protocols, or MPI_MSD. P values were calculated using a paired two-tailed t test.

We hypothesize that RECON is able to operate at higher efficiency by restricting sampled sequences to more relevant sequence space. We further believe that our conception of “relevant” sequence space is reflected in an ensemble of biologically observed sequences, and that RECON should recover not only a native protein sequence, but also biologically tolerated mutations. To address this question we generated a position-specific scoring matrix (PSSM) of amino acid frequencies in evolutionarily related proteins to each benchmark protein using a PSI-Blast query [25]. Among the promiscuous proteins we restricted this analysis to non-antibodies, since the full-length sequence of a mature antibody is unlikely to have a large number of meaningful evolutionary counterparts. However, since antibodies in the common germline-encoded benchmark set were only designed in positions deriving from the V_H_ gene, we were able to derive a PSSM from other common V_H_-encoded antibodies in the database. We then compared the PSSM to the amino acid frequency in corresponding positions in designed sequences to estimate how well the design protocol mimicked evolution. We measured agreement of sequence profiles using a modified Sandelin-Wasserman similarity to yield a percent similarity for each designed position that could then be averaged over the protein [26]. Fig 5A shows a comparison of positions in the V_H_5-51 benchmark where designs either agreed or disagreed with evolutionary sequence profiles—the degree of agreement could then be quantitated by the percent similarity calculated over each position.

### Comparison of designs to observed sequence profiles

We found that RECON was able to create sequences that more closely mirrored natural sequence variation than MPI_MSD (Table 6 and Fig 5B). Averaging over the benchmark cases, we observed 69, 73, and 57% similarity to evolutionary sequence profiles using RECON fixed backbone, backbone minimized, and MPI_MSD, respectively. This pattern was especially strong in benchmark cases with large numbers of designable residues, as the number of designed residues correlated positively with the improvement of RECON over MPI_MSD in recapitulating evolutionary sequence profiles (Fig 5C). When comparing the four largest benchmark cases by number of designable residues (three common germline-derived antibodies and the PAPD complex), RECON shows a marked improvement over MPI_MSD in recovery of evolutionary sequence profile (Fig 5D). Although this result is not significant due to a small sample size, it is suggestive of the additional benefit provided by RECON when applied to large, computationally intensive design problems. We hypothesize that this is due to compressed sequence space explored by RECON. When design problems are relatively small, the genetic algorithm employed by MPI_MSD is able to efficiently search through sequence space for a low-energy solution. However, when the sequence space increases in a large design problem the compressed sequence search is more advantageous.

**Table 6:** Comparison of design-generated sequences to evolutionary sequence profiles of input proteins.

	Evolutionary sequence similarity (%)^a^		
Benchmarkcase	RECON FBB	RECON BBM	MPI_MSD
**CheY**	56.3	70.5	57.5
**Elastase**	60.3	70.7	65.9
**FYN**	87.0	87.0	96.0
**PAPD**	61.7	65.3	52.4
**Ran**	76.6	79.3	82.5
**V**_**H**_ **1-69**	90.6	91.7	32.0
**V**_**H**_ **3-23**	50.7	50.7	36.4
**V**_**H**_ **5-51**	69.0	67.0	30.4
**Average**	**69.0**	**72.8**	**56.6**

Designs produced by MPI_MSD or fixed backbone (FBB) or backbone minimized (BBM) RECON algorithms were compared to sequence profiles of evolutionarily related proteins at designed positions.

^a^Sequence similarity is computed as the Sandelin-Wasserman similarity, normalized as a percentage. See methods for details.

### RECON searches a compressed, more relevant sequence space

We have shown that designs generated by RECON tend to more closely represent the evolutionary sequence profiles of our benchmark proteins when compared with MPI_MSD. We propose that this is accomplished via a more focused sequence search within the biologically relevant space. To further support this claim, we have analyzed the sequence space searched by RECON and MPI_MSD and compared it to the final output sequences of the top ten designs for the V_H_5-51 benchmark set (S1 Fig). We generated the sequence space profile by including any residue that was sampled at any step of the design protocol at each position, and then compared this profile to the final sequences among the top ten designs. Presumably the most efficient algorithm would only sample the sequences that are eventually selected as low energy solutions, resulting in a similarity of 100% between sequence space explored and output designed sequences. Therefore we used this similarity as an indicator of the degree of “wasted” sequence space, which is explored but never part of a low energy solution. Comparison of the profiles generated by RECON on a fixed backbone and MPI_MSD show that RECON explores space much more closely constrained to the final low energy sequences, with a similarity score of 92%, as compared to 80% for MPI_MSD. This further supports the claim that RECON searches a compressed search space to encounter a low energy multi-specific solution.

### Structural differences in residues preferred by different algorithms

**Fig 6:**
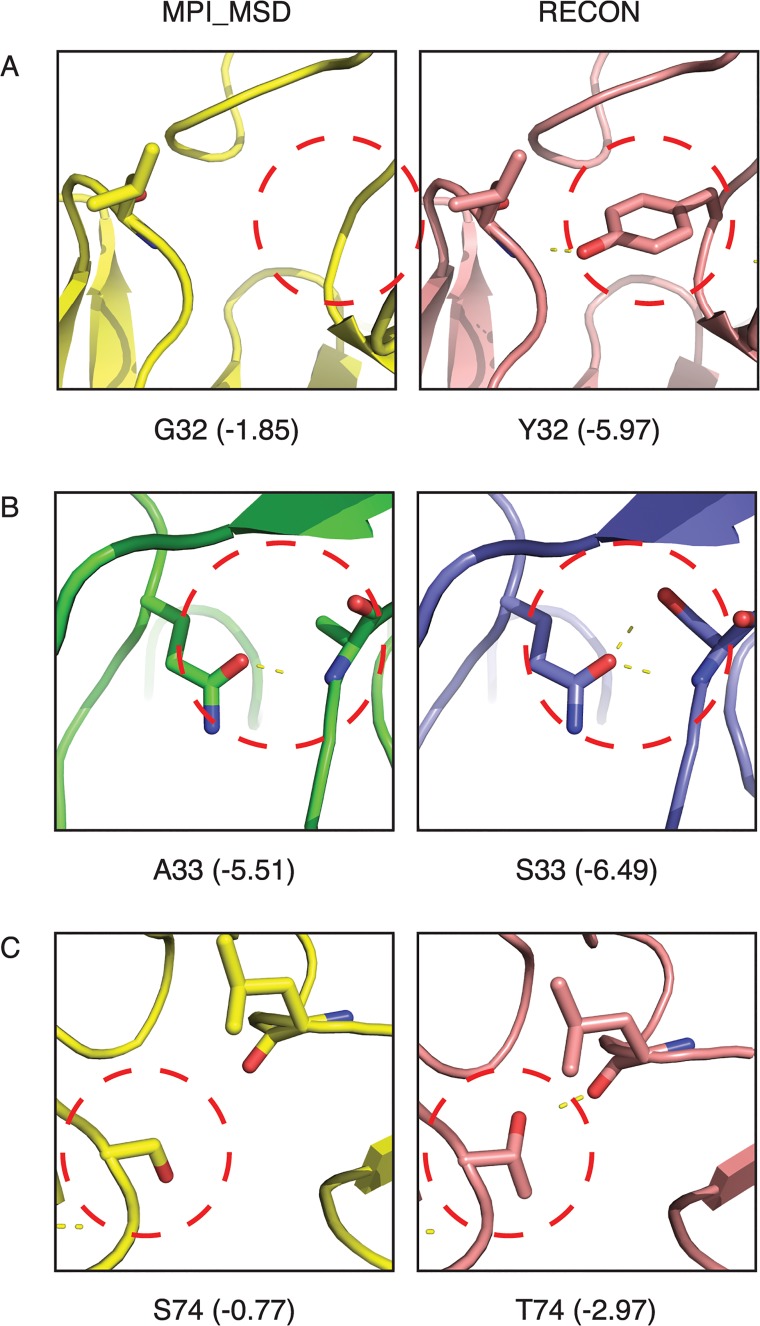
Structural analysis of sequence preferences of RECON and MPI_MSD. At positions 32 (A), 33 (B), and 74 (C), RECON and MPI_MSD showed consistent difference in sequence preference in the V_H_3-23 benchmark. Circled in red are positions that differ between the two structures. Shown in parenthesis are per-residue energy scores in REU summed across all post-minimization states. Shown above are post-minimization structures from designs generated by RECON and MPI_MSD. Structures shown in panels A and C are from the 1S78 complex, and those in panel B are from the 3BN9 complex.

The algorithms RECON and MPI_MSD feature substantial differences in sequence and structure at many positions of the output design models, particularly in the common germline antibody benchmark sets. We hypothesized that this difference in preference may be due to a failure by MPI_MSD to exhaustively search through sequence space in a large design problem. Concurrently we expect that the sequences selected for by RECON are actually lower in overall fitness. We present structural analysis of three positions, residues 32, 33, and 74 in the V_H_3-23 benchmark, to support this claim. Position 32 showed a preference for tyrosine in RECON-generated designs, whereas MPI_MSD prefers glycine. Tyrosine is able to fill a cross-interface gap in the 1S78 complex, and can establish hydrogen bonding to an amide nitrogen across the interface (Fig 6A). This additional hydrogen bonding produces a large drop in fitness for this residue across all states (-1.85 versus -5.97 REU). Interestingly, tyrosine is the germline residue at this position, and was only recovered using RECON with backbone minimization—both RECON fixed backbone and MPI_MSD favor glycine at this position. Position 33 also showed difference preferences between design methods—alanine was favored by MPI_MSD, whereas RECON favored serine. Serine results in a lower overall fitness due to additional hydrogen bonding with a glutamine residue on the heavy chain CDR3 loop of the antibody (Fig 6B). At this position, alanine is the germline residue—however the per-residue fitness values indicate that serine is able to stabilize this loop in the 3BN9 complex without compromising stability of the other states (Fig 6B, fitness shown in parenthesis). Lastly, position 74 showed a preference for threonine in RECON-generated designs, as opposed to serine in MPI_MSD-generated designs. Threonine is able to establish cross-interface hydrogen bonding in the 1S78 complex without causing clashes in other states, whereas serine is somewhat surprisingly not positioned to create this interaction (Fig 6C). This is partially due to backbone movements in the RECON-generated structure that position the hydroxyl group for optimal hydrogen bond geometry. In addition to hydrogen bonding, threonine scores more favorably on the basis of increased van der Waals attractive forces of the additional methyl group with surrounding atoms. At this position, asparagine is the germline amino acid, which was recovered by neither RECON nor MPI_MSD.

### Incorporating backbone motion results in increased recapitulation of evolutionary sequence profile

**Fig 7:**
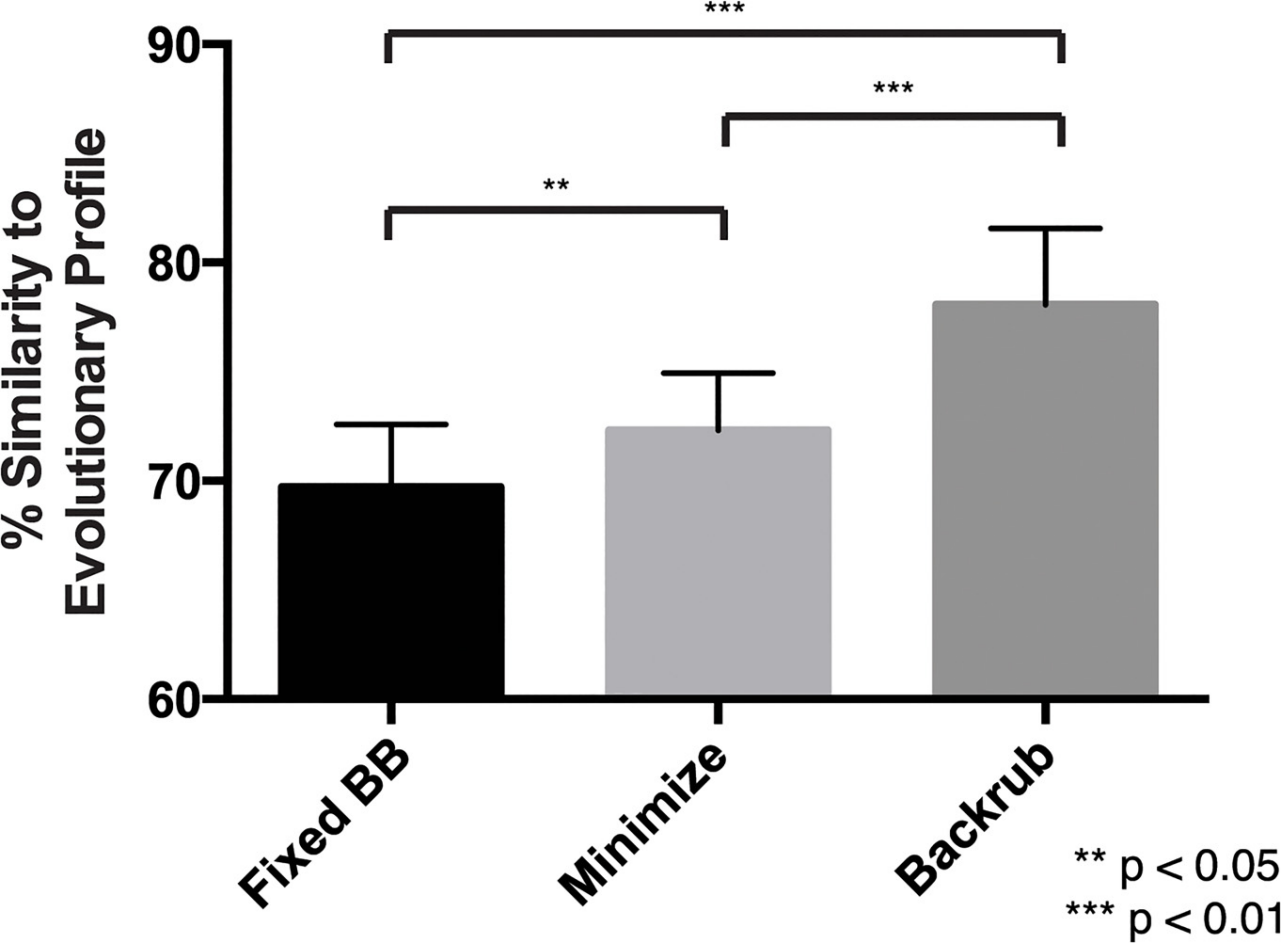
Incorporation of backbone motion into RECON recapitulates evolutionary sequence profiles in un-minimized structures. Multi-specificity design using RECON was repeated on structures that had not been previously energy minimized to evaluate the benefit of incorporating backbone movements. Designs were generated using either a fixed backbone protocol (Fixed BB), alternating rounds of φ, ψ, and χ angle minimization (Minimize), or using backrub motions (Backrub). P values were calculated by a paired two-tailed t test.

From our initial benchmark results, we did not observe a difference in evolutionary sequence similarity for designs created with fixed backbone versus backbone minimization protocols (Fig 5D). However, as previous reports have shown the utility of incorporating backbone motion into a design protocol [8,27–29], we hypothesized that the initial minimization of structures before entering them into multi-specificity design reduced the impact of alternating backbone minimization with design. We hypothesize that backbone movement should have a larger impact on design of structures that have not been pre-minimized. To test this hypothesis, we repeated the benchmark with structures that had not been pre-minimized, and performed multi-specificity design with three protocols: 1) fixed backbone design, 2) alternating design with minimization of φ, ψ, and χ angles, and 3) alternating design with backrub movements. The backrub motion involves rotation of a rigid backbone around axes between nearby Cα atoms, and has been shown to recapitulate alternative backbone conformations in high-resolution crystal structures [30] as well as improving prediction of the conformation of point mutant side chains [31]. We predicted that a design protocol including backrub motions between design rounds should result in the highest agreement to evolutionary sequence profiles, given the sampling of more biologically relevant conformational space than simple minimization. We therefore analyzed the similarity to evolutionary sequence profiles for the top ten designs produced by the three methods and compared to evaluate whether backbone motion in this context confers any additional benefit. As expected, incorporating backrub movements results in a statistically significant increase in similarity to evolutionary profiles as compared to a fixed backbone protocol or one involving minimization (Fig 7). This agrees with previous studies indicated that backrub motions are able to sample biologically relevant conformational space, and shows that backrub motions can be incorporated in a multi-specificity context to provide more robust results in terms of evolutionary sequence recovery.

### Advantage of multi-specificity vs. single-state design

In previous works involving both germline antibodies and promiscuous proteins, the difference in sequence recovery between sequences generated by single state and multi-specificity design has been analyzed [14,19]. Multi-specificity design in both cases was shown to recover the native or germline sequence at a higher rate than single state design, supporting the proposition that the increased performance of multistate design justifies the increased computational complexity. Given the increased performance of RECON in native sequence recovery, we hypothesized that multi-specificity design performed by RECON would result in a larger difference in germline vs. mature sequence recovery in the common germline antibody dataset. We therefore performed fixed backbone single state design for each complex in this dataset and calculated recovery of the germline sequence and the mature antibody sequences. We can recover the difference in germline and mature sequence recovery as observed in [19], and show that design performed by RECON results in a larger difference between germline and mature sequence recovery compared to MPI_MSD (S2 Fig). We can therefore conclude that in these cases RECON is more robust at generating germline-like, multi-specific sequences compared to MPI_MSD.

## Discussion

### Summary of results

We have developed and benchmarked a new method for multi-specificity design, REstrained CONvergence in multi-specificity design (RECON). This algorithm operates by allowing each state to search sequence space independently with a restraint system that gradually encourages convergence between different states on a common sequence. Allowing each state to adopt a unique sequence reduces the space of sequences required to search in order to find a native-like low energy solution. In two separate benchmark sets consisting of ten total cases, we were able to show that RECON, both with and without iterative backbone minimization cycles, was able to more accurately recapitulate the native, multi-specific sequence of input proteins than the existing MSD application in Rosetta, MPI_MSD. In addition, we analyzed agreement of designed sequences with observed evolutionary sequence profiles to measure how well MSD simulates natural sequence tolerance. In large design problems with many residues being optimized simultaneously, RECON was able to create sequences that more closely mirrored the natural distribution of sequences seen in evolutionary profiles.

### Diversity in predicted sequence tolerance

In this study we analyzed the degree of convergence of a designed protein sequence profiles with the natural sequence variation seen in evolutionary homologs. It is well known that many proteins tolerate a wider variety of sequences than simply the native sequence [13,29,32,33]—therefore a major goal of multi-specificity design is to recover not only the native sequence of a protein, but also sequence variations that are tolerated by all binding partners. We found that RECON is able to recover evolutionary sequence profiles more effectively in large complexes—however, it is clear from analysis of sequences sampled by each method (S1 Fig) that MPI_MSD is exploring a much larger sequence space. In certain cases this diversity of sampling may be desired, especially in cases where the interface with both binding partners is compatible with a large number of sequence polymorphisms. Our benchmark cases suggest that sampling near the energy minimum for each individual state is sufficient to recover the sequences compatible with all states. However, in cases where generating sequence diversity is at a premium, for example to explore the tolerated sequence space of a given backbone, it may be advantageous to use RECON and MPI_MSD as complementary approaches.

### Comparison of rotamer packing algorithms

RECON in the current study was used with the standard Rosetta simulated annealing rotamer optimization protocol [34]–however, other rotamer optimization methods have shown superior performance in certain instances. For example, MPI_MSD uses a modified form of the FASTER algorithm [35], referred to as backbone-minimum-energy conformation followed by single-residue perturbation/relaxation (BMEC-sPR) [15]. Leaver-Fay et al. compared the effectiveness of these two algorithms and found that BMEC-sPR consistently reached the global minimum solution in a higher proportion of cases [15]. Additional rotamer optimization algorithms have been adapted for use in MSD, such as dead-end elimination [36], probabilistic graphical models [37], and iterative batch relaxation/single perturbation and relaxation [35]. The benefit of RECON is that it can be adapted to work with any single state-compatible rotamer optimization method, as communication between different states is conducted solely by the restraint system. This opens up the possibility of adapting many more optimization methods for MSD.

### Fixed backbone versus backbone flexibility in restrained multi-state design

One important benefit of RECON is the ability to incorporate backbone motion into an MSD protocol. Traditionally protein flexibility in MSD has been modeled by including multiple backbone conformations as input states [9,13,16,32,38]. This is a reasonable strategy for running MSD using RECON. However, RECON offers the benefit that each state can be subject to additional backbone minimization between design rounds. When incorporating backbone motion into design the conformational and sequence space explodes, making it difficult to reach a global minimum. However the fact that RECON reduces the sampling needed to reach the optimal sequence allows for more search space to be explored. We have shown that incorporating backbone flexibility in the form of backrub motions can improve accuracy of sequences when applied to un-minimized structures. Single state design protocols have successfully incorporated backbone movement, allowing the introduction of mutations that would have been unfavorable on the original backbone [1,8,27]. The ideal protocol for flexible backbone design remains elusive, considering the different methods of backbone perturbation [28,30]. In addition it remains unclear how to best alternate fixed backbone sequence optimization with backbone motion [39]. RECON opens up the possibility of incorporating these backbone design methods into an MSD context.

### Negative design capabilities

One of the most challenging aspects of MSD is the inclusion of unfavorable states to destabilize. The current implementation of RECON is limited in scope compared to approaches such as MPI_MSD due to the inability to perform negative design to disfavor certain states. This limitation is not fundamental as in principle unfavorable states could be designed against with an energy penalty. However, it is outside the scope of the current work to benchmark such an approach. MSD has been successful in engineering proteins when both including [6,7,10,12] and ignoring [9,13,38] these negative design states. Bolon et al. have shown that including negative states produces designs that exhibit better specificity between competing states [40]–however, this comes at the cost of target protein stability [22,40], and therefore may not be ideal for every design problem. In addition, negative design states result in a significantly more complicated computational protocol–differences between backbone conformations can cause failures in rotamer placement that lead to artificially high energies [15]. This complicates the inclusion of multiple backbone states in an MSD problem, which mimics the natural flexibility of a protein in solution and results in higher quality designs [9,13,16,38]. Explicit negative design is not currently supported using RECON–the lack of an explicit fitness function makes it difficult to reconcile energies of positive states with negative ones. Grigoryan et al. used an intriguing “specificity sweep” protocol that alternates design rounds optimizing stability of positive states with specificity rounds, accepting mutations that destabilize the negative states without a negative effect on the positive ones [10]. A similar strategy could incorporate RECON to optimize stability and specificity without explicitly designing against a negative state.

### Integration of restrained multi-state design into Rosetta code base

RECON was designed with the intent to be easily integrated into the RosettaScripts computational framework [41]. To this end we emphasize that RECON is compatible with any other protocol that is available within RosettaScripts, which is not available for MPI_MSD. This makes it easier for users with experience running SSD protocols in RosettaScripts to expand their capabilities by including RECON. This can be used to include additional conformational states, explicitly model bound and unbound conformations, or simultaneously design against multiple partners. We describe in the supplemental materials (S1 File) a comprehensive example of how to run RECON in an XML file.

## Methods

### Selection of datasets for benchmark

Common germline gene-derived antibody complexes were selected and processed as in [19]–briefly, candidate complexes were selected by querying the Immunogenetics Information System (IMGT) 3D structural query system for antibodies derived from either V_H_1-69, V_H_3-23, or V_H_5-51 germline genes [21]. Only complexes containing protein or peptide ligands were considered. Common germline antibodies were only considered for multi-specificity design when derived from the same allele. Promiscuous proteins used were derived from the multi-specificity design study described in Humphris et al. 2007 [14]. Complexes were selected to maximize diversity of structure and function, as well as to select proteins with diverse ligands.

### Preparation of structures for design simulations

Structures were downloaded from the Protein Data Bank (PDB; www.rcsb.org), and manually processed to remove water and non-proteinogenic molecules. Any chain breaks were closed using kinematic loop closure [42]. Due to extensive chain breaks in CDR loops, chains H and L in structure 3GBM were replaced by the same chains in 3GBN. Structures were subject to energy minimization in Rosetta using the talaris 2013 score function [17]. The lowest energy model of 50 energy-minimized models for each complex was selected for design.

### Multi-specificity design

For common germline antibody multi-specificity design, amino acid sequences deriving from the V_H_ gene were aligned using ClustalW sequence alignment [43], and positions that varied in any of the antibodies were specified for design. Germline sequences were inferred from IMGT/3D Structure DB [21]. Designable residues in promiscuous proteins were selected as those present in the interface of all binding partners. To define interface residues, a set of filters was applied to select residues that are likely to engage in interactions with the opposing chain. The first filter eliminates any residue with a Cβ distance larger than 10 Å from the closest residue in the opposing chain. Residues were then selected that either had a heavy atom within 5.5 Å of a heavy atom across the interface, or those with an angle of less than 75° between two vectors, Cα-Cβ of the residue and Cβ-Cβ to the closest residue Cβ on the opposing chain. This vector angle filter allows inclusion of residues where the sidechain is oriented to face the opposing chain. In addition, any residues at the interface on the side of the binding partner were specified for repacking. Identical residues for design and repacking were used for both RECON and MPI_MSD. For RECON benchmarking, fixed backbone design was used with 4 rounds of rotamer packing. RECON constraints were ramped through the 4 rounds of design using convergence restraints of 0.5, 1.0, 1.5, and 2.0 REU. Sequence convergence was enforced at the end of the protocol using a greedy selection algorithm. RECON was also benchmarked with backbone minimization–this protocol was identical to the fixed backbone protocol with the addition of a cycle of minimization of φ, ψ, and χ angles after each design round. At the end of the backbone minimization protocol we performed one full round of a Rosetta relax protocol, which involves rotamer packing and minimization using a gradually increasing repulsive force [44]. In simulations performed with backrub motions, all backbone atoms on the protein chain being designed were specified as pivot residues. The backrub motion as implemented in Rosetta is described in detail in [31]. MPI_MSD was run with default parameters, with the number of rounds defined as 15 times the number of designable residues [15]. For MPI_MSD, the fitness function was defined as the sum of energy of the complexes. Single state design was run as four rounds of fixed backbone rotamer optimization, using the same designable and repackable residues as previously specified. The talaris 2013 scoring function was used for all methods of design.

### Quantitative measures for analysis of resulting sequences

For each complex, 100 designs were created as described using both RECON and MPI_MSD applications. Sequence logos were created using the Berkeley web logo server (http://weblogo.berkeley.edu). Bitscore was computed for each design trajectory, defined as the Shannon entropy of each amino acid occurring at each designed position, described in [19] [45,46]. Bitscore was calculated using the following equation: I_i_ = p_i_ x log_2_(20 x p_i_), where i represents the amino acid and p_i_ is the frequency in the top ten designs. When p_i_ is 100% the bitscore becomes 4.32, which was used as the maximum possible bitscore in our calculations. To calculate native sequence recovery, the summed bitscore of the native amino acid at each position was divided by the sum of the bitscore of all amino acids at all positions. Designs were analyzed on the basis of the fitness of the top ten designs, with fitness defined as the sum of Rosetta energy of all states, and native sequence recovery. Rosetta energy was reported in all cases with convergence restraints subtracted from the total.

To generate an evolutionary sequence profile we used PSI-Blast with default parameters, querying a non-redundant protein database [25,47]. This position-specific scoring matrix (PSSM) of amino acid frequencies was then compared to a PSSM constructed from observed frequencies in the top ten designs by fitness resulting from RECON or MPI_MSD. To compare PSSMs we used a modified Sandelin-Wasserman similarity score [26]. This score was calculated by computing the squared difference for each amino acid frequency at each position. The squared difference was summed for all amino acids at a given position, and subtracted from two to yield a similarity score from 0 (no similarity) to 2 (identical). This value was then normalized by a factor of two to yield a percent similarity for each position and summed over all designed positions to give an overall similarity score. To reduce background noise when comparing PSSMs we only compared positions that had any observed mutations in the top ten designs produced by any design method. Inclusion of positions where no mutations are observed would inflate evolutionary similarity values for all methods. This reduced the total number of positions considered from 200 among eight benchmark sets to 97. In the benchmark cases of un-minimized structures, eliminating positions with no variation by any method left 151 of 200 possible positions.

## Supporting Information

S1 Table Post-minimization fitnesses of benchmark sets. Structures generated by design were energy minimized to relieve small clashes. Fitnesses reported are the sum of energy of all states. Best values in each row are shown in bold.
(DOCX)

S2 Table Performance of a control greedy selection algorithm. Design of benchmark cases was repeated for a greedy selection algorithm, which lacks the ramping convergence restraints of RECON. This algorithm performs a single round of unrestrained design followed by a greedy selection of amino acids that maximize fitness over all states.
(DOCX)

S3 Table Non-converging positions in the V_H_5-51 benchmark set. Failure to converge by the end of the RECON convergence restraint protocol was counted for each designed residue in the V_H_5-51 benchmark set, over 100 design trajectories.
(DOCX)

S1 Fig The sequence space explored by RECON and MPI_MSD is compared to the sequence profiles of the top ten designs for the V_H_5-51 benchmark case. Sequence space was determined as any amino acid that was sampled at any point throughout the design protocol. A similarity score was calculated between the sequence space explored by an algorithm and the top ten designs produced by the same algorithm.
(EPS)

S2 Fig Germline and mature sequence recovery for sequences generated by RECON and MPI_MSD multi-specificity design, compared to the sequences generated by single state design. (EPS)

S1 File Protocol capture file containing in-detail description of the computational methodology, as well as scripts useful in generating input files and analyzing results. (PDF)
