# Supplementary material for: Design of Protein Multi-specificity Using an Independent Sequence Search Reduces the Barrier to Low Energy Sequences
Source: PLoS Comput Biol. 2015 Jul 6;11(7):e1004300. doi: 10.1371/journal.pcbi.1004300 (PMC4493036; doi:10.1371/journal.pcbi.1004300)
Supplement: S1 Table — Structures generated by design were energy minimized to relieve small clashes. Fitnesses reported are the sum of energy of all states. Best values in each row are shown in bold. (DOCX) [file pcbi.1004300.s001.docx]

**Table S1.** Post-minimization fitnesses of benchmark sets.

|  | **Post-minimization fitness (REU)** | | |
| --- | --- | --- | --- |
| **Protein/Germline**  **gene** | **RECON FBB** | **RECON**  **BBM** | **MPI_MSD** |
| **CheY** | -1113.5 | **-1119.7** | -1119.2 |
| **CR6261** | -2532.6 | **-2537.7** | -2532.0 |
| **Elastase** | -1445.4 | -1445.1 | **-1447.9** |
| **FI6v3** | -2506.0 | **-2515.2** | -2506.2 |
| **FYN** | -777.2 | **-780.3** | -778.3 |
| **PapD** | -1903.5 | -1891.4 | **-1908.8** |
| **Ran** | -3675.2 | -3716.4 | **-3755.8** |
| **V_H_1-69** | -5299.1 | -5306.7 | **-5343.5** |
| **V_H_3-23** | -3410.0 | -3427.1 | **-3479.9** |
| **V_H_5-51** | -2329.4 | -2348.3 | **-2360.5** |

Structures generated by design were energy minimized to relieve small clashes. Fitnesses reported are the sum of energy of all states. Best values in each row are shown in bold.
