# Supplementary material for: Design of Protein Multi-specificity Using an Independent Sequence Search Reduces the Barrier to Low Energy Sequences
Source: PLoS Comput Biol. 2015 Jul 6;11(7):e1004300. doi: 10.1371/journal.pcbi.1004300 (PMC4493036; doi:10.1371/journal.pcbi.1004300)
Supplement: S2 Table — Design of benchmark cases was repeated for a greedy selection algorithm, which lacks the ramping convergence restraints of RECON. This algorithm performs a single round of unrestrained design followed by a greedy selection of amino acids that maximize fitness over all states. (DOCX) [file pcbi.1004300.s002.docx]

**Table S2.** Performance of a control greedy selection algorithm.

|  | **Native sequence recovery (%)** | | **Fitness**  **(REU)** | |
| --- | --- | --- | --- | --- |
| **Protein/Germline**  **gene** | **RECON FBB** | **Greedy selection** | **RECON FBB** | **Greedy selection** |
| **CheY** | 80.6 | 60.0 | -1093.1 | -825.1 |
| **CR6261** | 79.0 | 69.2 | -2499.5 | -1531.7 |
| **Elastase** | 84.8 | 80.0 | -1383.8 | -767.5 |
| **FI6v3** | 57.8 | 44.1 | -2459.1 | -1912.4 |
| **FYN** | 100.0 | 100.0 | -758.3 | -712.3 |
| **PapD** | 92.5 | 65.7 | -1685.5 | -1241.4 |
| **Ran** | 87.1 | 73.9 | -2682.3 | -2637.4 |
| **V_H_1-69** | 94.2 | 59.0 | -3015.9 | -2314.6 |
| **V_H_3-23** | 55.3 | 27.2 | -911.7 | 939.2 |
| **V_H_5-51** | 65.9 | 40.1 | -840.5 | 50.6 |
| **Average** | **79.7** | **61.9** | **-1733.0** | **-1095.3** |

Design of benchmark cases was repeated for a greedy selection algorithm, which lacks the ramping convergence restraints of RECON. This algorithm performs a single round of unrestrained design followed by a greedy selection of amino acids that maximize fitness over all states.
