# Supplementary material for: Design of Protein Multi-specificity Using an Independent Sequence Search Reduces the Barrier to Low Energy Sequences
Source: PLoS Comput Biol. 2015 Jul 6;11(7):e1004300. doi: 10.1371/journal.pcbi.1004300 (PMC4493036; doi:10.1371/journal.pcbi.1004300)
Supplement: S3 Table — Failure to converge by the end of the RECON convergence restraint protocol was counted for each designed residue in the VH5-51 benchmark set, over 100 design trajectories. (DOCX) [file pcbi.1004300.s003.docx]

**Table S3.** Non-converging positions in the V_H_5-51 benchmark set.

| **Position** | **Converging count** | **Non-converging count** |
| --- | --- | --- |
| 2 | 100 | 0 |
| 5 | 100 | 0 |
| 14 | 0 | 100 |
| 16 | 100 | 0 |
| 23 | 100 | 0 |
| 24 | 0 | 100 |
| 29 | 0 | 100 |
| 30 | 100 | 0 |
| 31 | 100 | 0 |
| 32 | 52 | 48 |
| 34 | 100 | 0 |
| 40 | 100 | 0 |
| 46 | 100 | 0 |
| 48 | 0 | 100 |
| 51 | 1 | 99 |
| 52 | 0 | 100 |
| 54 | 100 | 0 |
| 58 | 1 | 99 |
| 65 | 100 | 0 |
| 70 | 100 | 0 |
| 72 | 100 | 0 |
| 74 | 100 | 0 |
| 76 | 96 | 4 |
| 77 | 100 | 0 |
| 80 | 0 | 100 |
| 84 | 100 | 0 |
| 88 | 100 | 0 |
| 93 | 100 | 0 |
| 97 | 100 | 0 |
| 98 | 0 | 100 |
| **Number of positions** | **21** | **9** |
| **Germline sequence recovery (%)** | **66.1** | **74.0** |

Failure to converge by the end of the RECON convergence restraint protocol was counted for each designed residue in the V_H_5-51 benchmark set, over 100 design trajectories.
