# Supplementary material for: Design of Protein Multi-specificity Using an Independent Sequence Search Reduces the Barrier to Low Energy Sequences
Source: PLoS Comput Biol. 2015 Jul 6;11(7):e1004300. doi: 10.1371/journal.pcbi.1004300 (PMC4493036; doi:10.1371/journal.pcbi.1004300)
Supplement: S1 File — (PDF) [file pcbi.1004300.s006.pdf]

Protocol Capture

Intro

The following is a detailed description of how to run and analyze the results from restrained convergence in multi-specificity design (RECON). RECON is used for multi-specificity design, to minimize the energy of multiple protein complexes simultaneously. This is useful in many different contexts, for example to design a single sequence that can bind multiple proteins with high affinity, or a sequence that stabilizes a protein in multiple conformations. In the benchmark cases we selected, the goal was to optimize the sequence of proteins with multiple binding partners to increase affinity for all binding partners. Below is a description of how to perform multi-specificity design of a protein, FYN kinase, which has been crystallized with two binding partners, and optimize the sequence to have low energy in complex with its two partners.

RECON is run completely within ROSETTASCRIPTS, as a combination of movers written specifically for the purpose of multistate design. This offers the benefit of making all other movers available within ROSETTASCRIPTS compatible with a multi-specificity protocol, i.e. backbone minimization, rigid body docking, atom pair constraints, etc. **All ROSETTA commands were run with version 8641cc1735a37dff08c3f1857bbe3035908f7f04.** All analysis scripts are available for download at https://github.com/sevya/msd_analysis_scripts. Note: scripts provided in the analysis directory are dependent upon each other, and when moved from this directory may not function properly.

PDB Preparation

First, the PDB structures were downloaded from the RCSB and manually inspected to remove all but one asymmetric unit. In this case, the PDB IDs of the FYN kinase structure of interest are 1AVZ and 1M27. Structures can be processed manually or with the clean_pdbs.py script, located in /path/to/Rosetta/tools/protein_tools/scripts/clean_pdb.py. This script will download the specific chains of your structure and remove all non-proteinogenic molecules, which makes the structure compatible with ROSETTA. The syntax for this command is:

clean_pdb.py 1avz ABC

clean_pdb.py 1m27 ABC

In this case chain C in both 1avz and 1m27 is the FYN kinase that will be designed. However 1m27 has an extra leading valine residue at the N-terminus that is not present in 1avz. To simplify the protocol this residue was removed in PyMol before proceeding - this residue can also be removed using a text editor. Next, the chains in each structure were reordered to put the one protein common to both structures, FYN, as the first chain, chains were renamed to A, B, and C, and each chain was renumbered starting from one. This simplifies the protocol by giving the input structure a common format. The renumbering can be done manually or with the script reorder_pdb_chains.py, which

takes as input your desired chain order, desired chain ids, and the input and output PDBs. This script simply moves the order of the chains to the desired order and renames the chains, while also renumbering residues from 1 to N. Note that this does not change the coordinates of any atoms, only the order in the PDB file and the chain identifier. The command for this is:

```
reorder_pdb_chains.py --new_chain_order C,A,B --new_chain_ids A,B,C 1avz.pdb
1avz_renum.pdb
```

```
reorder_pdb_chains.py --new_chain_order C,A,B --new_chain_ids A,B,C 1m27.pdb
1m27_renum.pdb
```

Next 50 relaxed models were created from each of the two starting PDBs, using the following commands, XML scripts and flags. Of the 50 relaxed models I selected the lowest energy model for the design process. The flags I use control the memory usage when ROSETTA is building side chain rotamers (linmem_ig), the number of extra rotamers to include in the library (ex1/2, use_input_sc), the number of models to make (nstruct), and a designation to include all side chain atoms (fullatom). For more information on ROSETTA relax and available options see https://www.rosettacommons.org/docs/latest/prepare-pdb-for-rosetta-with-relax.html. Below are the commands used to create relaxed models.

```
/path/to/Rosetta/main/source/bin/rosetta_scripts.default.linuxgccrelease @relax.flags -s
1avz_renum.pdb -parser:protocol relax.xml
```

relax.xml:

```
<ROSETTASCRIPTS>
   <SCOREFXNS>
   </SCOREFXNS>
   <TASKOPERATIONS>
      <InitializeFromCommandline name=ifcl />
      <RestrictToRepacking name=rtr />
   </TASKOPERATIONS>
   <FILTERS>
   </FILTERS>
   <MOVERS>
      <FastRelax name=relax repeats=8 task_operations=ifcl,rtr
min_type=lbfgs_armijo_nonmonotone/>
   </MOVERS>
   <APPLY_TO_POSE>
   </APPLY_TO_POSE>
   <PROTOCOLS>
        <Add mover=relax />
   </PROTOCOLS>
```

```
</ROSETTASCRIPTS>
```

relax.flags:

```
-database /path/to/Rosetta/main/database
-linmem_ig 10
-ex1
-ex2
-in:file:fullatom
-out:file:fullatom
-use_input_sc
-nstruct 50
```

Input files

Once the input structures have been processed, the input files needed for RECON can be generated. First residue files (resfiles) are needed that specify the designable and repackable residues for both of my complexes. Residues that are designable can be substituted with any other amino acid, whereas ones that are repackable can only be substituted with different rotational isomers (rotamers) of the same amino acid. For more information on resfile syntax and logic see https://www.rosettacommons.org/manuals/archive/rosetta3.5_user_guide/d1/d97/resfiles.html. In this case all residues on chain A that are at the interface of chain A and chains B+C were chosen for design. Since the two complexes have different binding partners they may have overlapping but not identical interface residues – in this case I selected only interface residues common to both complexes. In addition I want to repack any residues on chains B+C that are at the interface. A residue file is needed for each complex that specifies which residues are to be designed and repacked. The number of designable residues must be the same between copmlexes, but repackable residues can be unique to each complex. The following script and flags will generate these files:

```
generate_interface_files.py --side1 A --side2 BC --design-side 1 --repack --output 1avz 1avz_relaxed.pdb
```

```
generate_interface_files.py --side1 A --side2 BC --design-side 1 --repack --output 1m27 1m27_relaxed.pdb
```

This identifies all residues at the interface between chains A and B+C, specifies side 1 as the one with designable residues, and signals to repack any residues at the opposing side of the interface. It also specifies a name for the output file, which will be followed by the extension .resfile. After generating residue files, to ensure that both complexes are designing the same number of residues it’s important to manually remove residues on the A chain that are at the interface of one complex but not the other. The resfiles used in the benchmark are shown below for reference:

1avz.resfile:

NATRO
start
12 A ALLAA EX 1 EX 2
13 A ALLAA EX 1 EX 2
14 A ALLAA EX 1 EX 2
15 A ALLAA EX 1 EX 2
16 A ALLAA EX 1 EX 2
35 A ALLAA EX 1 EX 2
48 A ALLAA EX 1 EX 2
1 C NATAA EX 1 EX 2
2 C NATAA EX 1 EX 2
3 C NATAA EX 1 EX 2
4 C NATAA EX 1 EX 2
5 C NATAA EX 1 EX 2
6 C NATAA EX 1 EX 2
7 C NATAA EX 1 EX 2
10 C NATAA EX 1 EX 2
12 C NATAA EX 1 EX 2
13 C NATAA EX 1 EX 2
16 C NATAA EX 1 EX 2
17 C NATAA EX 1 EX 2
20 C NATAA EX 1 EX 2
47 C NATAA EX 1 EX 2
48 C NATAA EX 1 EX 2
49 C NATAA EX 1 EX 2
50 C NATAA EX 1 EX 2

1m27.resfile:
NATRO
start
12 A ALLAA EX 1 EX 2
13 A ALLAA EX 1 EX 2
14 A ALLAA EX 1 EX 2
15 A ALLAA EX 1 EX 2
16 A ALLAA EX 1 EX 2
35 A ALLAA EX 1 EX 2
48 A ALLAA EX 1 EX 2
61 B NATAA EX 1 EX 2
63 B NATAA EX 1 EX 2
75 B NATAA EX 1 EX 2
76 B NATAA EX 1 EX 2
77 B NATAA EX 1 EX 2
78 B NATAA EX 1 EX 2
79 B NATAA EX 1 EX 2

82 B NATAA EX 1 EX 2
83 B NATAA EX 1 EX 2
85 B NATAA EX 1 EX 2
86 B NATAA EX 1 EX 2

RECON Script

The following script contains the RECON fixed backbone protocol:

```
<ROSETTASCRIPTS>
	<SCOREFXNS>
		<tal weights=talaris2013.wts >
			<Reweight scoretype=res_type_constraint weight=1.0 />
		</tal>
	</SCOREFXNS>
	<TASKOPERATIONS>
		<InitializeFromCommandline name=ifcl />
		<RestrictToRepacking name=rtr />
	</TASKOPERATIONS>
	<MOVERS>
		<PackRotamersMover name=design scorefxn=tal task_operations=ifcl />
		<MSDMover name=msd1 design_mover=design constraint_weight=0.5
resfiles=1avz.resfile,1m27.resfile debug=1 />
		<MSDMover name=msd2 design_mover=design constraint_weight=1
resfiles=1avz.resfile,1m27.resfile debug=1 />
		<MSDMover name=msd3 design_mover=design constraint_weight=1.5
resfiles=1avz.resfile,1m27.resfile debug=1 />
		<MSDMover name=msd4 design_mover=design constraint_weight=2
resfiles=1avz.resfile,1m27.resfile debug=1 />
		<FindConsensusSequence name=finish scorefxn=tal
resfiles=1avz.resfile,1m27.resfile />
	</MOVERS>
	<FILTERS>
	</FILTERS>
	<APPLY_TO_POSE>
	</APPLY_TO_POSE>
	<PROTOCOLS>
		<Add mover=msd1 />
		<Add mover=msd2 />
		<Add mover=msd3 />
		<Add mover=msd4 />
		<Add mover=finish />
	</PROTOCOLS>
</ROSETTASCRIPTS>
```

In this case the design mover used is a PackRotamersMover, which is given to each MSDMover as a submover. Note that the design mover is never actually called – it is called within the MSDMover. The four MSDMovers also specify a weight for residue constraints, which are ramped throughout the protocol, and a debug flag for extra output messages. The resfiles tag uses the files generated in the previous step to tell the MSDMover which residues should be linked together in multistate design. The resfiles don't need to have designable residues at the same positions (i.e. position 1 on protein 1 can correspond to a position 10 on protein 2), but they must have the same number of total designable residues. **Note: RECON matches resfiles to structures by input order. It is critical that PDBs are specified on the command line in the same order as resfiles in the XML file.** FindConsensusSequence is the greedy selection protocol to ensure that a single multi-specific sequence results from RECON. It checks at each position specified in the resfiles if the two input PDBs have a different amino acid, and if they do it places each of the candidate amino acids onto all states, packs rotamers and checks the sum of energy across states. Whichever of the candidates results in the lowest energy across all states is accepted as the final identity.

A flags file is also needed to specify ROSETTA options – the following are the flags used in the benchmark:

-in:file:fullatom
-out:file:fullatom
-database /path/to/Rosetta/main/database/
-linmem_ig 10
-ex1
-ex2
-nstruct 100
-run:msd_job_dist
-run:msd_randomize

The only flags specific to the RECON protocol are the last two. Run:msd_job_dist is needed for the JobDistributor to be able to give multiple input poses to a mover at the same time, which is needed for multi-specificity design. This protocol will fail and throw an error message without this flag. Run:msd_randomize randomizes the order of input poses before applying each mover. This is not completely necessary for multi-specificity design but is recommended, the reason being that there is slightly different behavior depending on the order in which PDBs are input. By randomizing the order before you keep this from biasing your results. More information on the other flags can be found at https://www.rosettacommons.org/docs/wiki/full-options-list.

Running RECON

Now that the setup is complete RECON can be performed with the following command line:

```
/path/to/Rosetta/main/source/bin/rosetta_scripts.default.linuxgccrelease @msd.flags -s
1avz.pdb 1m27.pdb -parser:protocol fix_bb.xml -scorefile fix_bb.fasc
```

This will generate 100 fixed backbone designs using RECON. For my backbone minimized designs the same options, input files, and commands were used, with the only difference being my xml:

```
<ROSETTASCRIPTS>
	<SCOREFXNS>
		<tal weights=talaris2013.wts >
			<Reweight scoretype=res_type_constraint weight=1.0 />
		</tal>
	</SCOREFXNS>
	<TASKOPERATIONS>
		<InitializeFromCommandline name=ifcl />
		<RestrictToRepacking name=rtr />
		<RestrictToInterfaceVector name=rtiv chain1_num=1 chain2_num=2,3
CB_dist_cutoff=10.0 nearby_atom_cutoff=5.5 vector_angle_cutoff=75
vector_dist_cutoff=9.0 />
	</TASKOPERATIONS>
	<MOVERS>
		<PackRotamersMover name=design scorefxn=tal task_operations=ifcl />
		<MSDMover name=msd1 design_mover=design constraint_weight=0.5
resfiles=1avz.resfile,1m27.resfile debug=1 />
		<MSDMover name=msd2 design_mover=design constraint_weight=1
resfiles=1avz.resfile,1m27.resfile debug=1 />
		<MSDMover name=msd3 design_mover=design constraint_weight=1.5
resfiles=1avz.resfile,1m27.resfile debug=1 />
		<MSDMover name=msd4 design_mover=design constraint_weight=2
resfiles=1avz.resfile,1m27.resfile debug=1/>
		<FindConsensusSequence name=finish scorefxn=tal
resfiles=1avz.resfile,1m27.resfile />
		<TaskAwareMinMover name=min tolerance=0.001 task_operations=rtiv
type=lbfgs_armijo_nonmonotone chi=1 bb=1 jump=1 scorefxn=talaris2013 />
		<FastRelax name=relax scorefxn=talaris2013 task_operations=ifcl,rtr,rtiv
repeats=1 />
	</MOVERS>
	<FILTERS>
	</FILTERS>
	<APPLY_TO_POSE>
	</APPLY_TO_POSE>
	<PROTOCOLS>
		<Add mover=msd1 />
		<Add mover=min />
		<Add mover=msd2 />
```

```
        <Add mover=min />
        <Add mover=msd3 />
        <Add mover=min />
        <Add mover=msd4 />
        <Add mover=min />
        <Add mover=finish />
        <Add mover=relax />
    </PROTOCOLS>
</ROSETTASCRIPTS>
```

In addition structures generated using backrub motions were generated using the same options, input files, and commands, with the following XML:

```
<ROSETTASCRIPTS>
    <SCOREFXNS>
        <tal weights=talaris2013.wts >
            <Reweight scoretype=res_type_constraint weight=1.0 />
        </tal>
    </SCOREFXNS>
    <TASKOPERATIONS>
        <InitializeFromCommandline name=ifcl />
    </TASKOPERATIONS>
    <MOVERS>
        <PackRotamersMover name=design scorefxn=tal task_operations=ifcl />
        <MSDMover name=msd1 design_mover=design constraint_weight=0.5
resfiles=%%resfiles%% debug=1 />
        <MSDMover name=msd2 design_mover=design constraint_weight=1
resfiles=%%resfiles%% debug=1 />
        <MSDMover name=msd3 design_mover=design constraint_weight=1.5
resfiles=%%resfiles%% debug=1 />
        <MSDMover name=msd4 design_mover=design constraint_weight=2
resfiles=%%resfiles%% debug=1/>
        <FindConsensusSequence name=finish scorefxn=tal
resfiles=%%resfiles%% />
        <BackrubDD name=brub moves=5000 >
            <span begin=1 end=57 />
        </BackrubDD>
    </MOVERS>
    <FILTERS>
    </FILTERS>
    <APPLY_TO_POSE>
    </APPLY_TO_POSE>
    <PROTOCOLS>
        <Add mover=msd1 />
```

```
        <Add mover=brub />
        <Add mover=msd2 />
        <Add mover=brub />
        <Add mover=msd3 />
        <Add mover=brub />
        <Add mover=msd4 />
        <Add mover=brub />
    </PROTOCOLS>
</ROSETTASCRIPTS>
```

MPI_MSD File Preparation

To run MPI_MSD the same designable and repackable residues were used, and files were reformatted for this application. Full documentation on the MPI_MSD application is available at https://www.rosettacommons.org/docs/latest/mpi-msd.html. Briefly, the necessary input files are described below. An entity resfile is needed that specifies the residues to be designed (FYN.entres), a correspondence file that maps designable residues to an index (FYN.corr), and secondary resfiles that specify which additional residues are to be repacked (1avz.2res, 1m27.2res). The residues included in these files are derived from the interface residues I used in RECON. In addition a fitness file is needed that specifies the fitness function used (fitness.daf), and state files for each input pdb (1avz.state, 1m27.state).

The contents of the input files used in the benchmark are shown below:

FYN.entres:

```
7
ALLAA EX 1 EX 2
start
1 A ALLAA EX 1 EX 2
2 A ALLAA EX 1 EX 2
3 A ALLAA EX 1 EX 2
4 A ALLAA EX 1 EX 2
5 A ALLAA EX 1 EX 2
6 A ALLAA EX 1 EX 2
7 A ALLAA EX 1 EX 2
```

FYN.corr:

```
1 12 A
2 13 A
3 14 A
4 15 A
5 16 A
6 35 A
```

7 48 A

1avz.2res:
NATRO EX 1 EX 2
start
1 C NATAA EX 1 EX 2
2 C NATAA EX 1 EX 2
3 C NATAA EX 1 EX 2
4 C NATAA EX 1 EX 2
5 C NATAA EX 1 EX 2
6 C NATAA EX 1 EX 2
7 C NATAA EX 1 EX 2
10 C NATAA EX 1 EX 2
12 C NATAA EX 1 EX 2
13 C NATAA EX 1 EX 2
16 C NATAA EX 1 EX 2
17 C NATAA EX 1 EX 2
20 C NATAA EX 1 EX 2
47 C NATAA EX 1 EX 2
48 C NATAA EX 1 EX 2
49 C NATAA EX 1 EX 2
50 C NATAA EX 1 EX 2

1m27.2res:
NATRO EX 1 EX 2
start
61 B NATAA EX 1 EX 2
63 B NATAA EX 1 EX 2
75 B NATAA EX 1 EX 2
76 B NATAA EX 1 EX 2
77 B NATAA EX 1 EX 2
78 B NATAA EX 1 EX 2
79 B NATAA EX 1 EX 2
82 B NATAA EX 1 EX 2
83 B NATAA EX 1 EX 2
85 B NATAA EX 1 EX 2
86 B NATAA EX 1 EX 2

fitness.daf:
STATE_VECTOR avz 1avz.state
STATE_VECTOR m27 1m27.state
FITNESS vmin(avz) + vmin(m27)

1avz.state:

1avz_relax_2_0010.pdb FYN_min.corr 1avz.2res

1m27.state:
1m27_relax_2_0003.pdb FYN_min.corr 1m27.2res

Running MPI_MSD

MPI_MSD can be run with the following command:

mpiexec -n 12 /path/to/Rosetta/main/source/bin/mpi_msd.default.linuxgccrelease -database /path/to/Rosetta/main/database/ -entity_resfile FYN.entres -fitness_file fitness.daf -ms::pop_size 100 -ms::generations 105 -ms::numresults 100 -no_his_his_pairE -ms::fraction_by_recombination .02 -msd::double_lazy_ig_mem_limit 100 -ex1 -ex2

This runs the application on 12 processors and generates 100 output files.

Design analysis

To perform design analysis structures are first sorted by the fitness of all designs, which is the sum of energy of my input proteins. I analyzed the top ten designs for each of these three methods for fitness, sequence recovery, and similarity to evolutionary sequence profile. After identifying the top ten designs I used the Weblogo server to generate sequence logos, and the deep_analysis script as a wrapper to calculate amino acid frequencies at each position and make my sequence logo. Deep analysis takes as input a resfile to identify which residues should be compared - however, note that a separate resfile should be made for only designable residues for this purpose (FYN_analysis.resfile), otherwise it will output a sequence logo for all designable and repackable residues. The contents of this resfile are shown below:

FYN_analysis.resfile:
start
12 A ALLAA EX 1 EX 2
13 A ALLAA EX 1 EX 2
14 A ALLAA EX 1 EX 2
15 A ALLAA EX 1 EX 2
16 A ALLAA EX 1 EX 2
35 A ALLAA EX 1 EX 2
48 A ALLAA EX 1 EX 2

Note: deep analysis does not link residues between complexes like RECON. It's most useful to analyze each input complex separately. However, since the result of my design run will be two complexes with exactly the same sequence at all designable positions, it's only strictly necessary to analyze sequences from one of the complexes. An example command for this script is the following:

deep_analysis --prefix 1avz_fixbb_ --native 1avz_renum.pdb --stack_width 30 --seq --format png --labels sequence_numbers --res FYN_analysis.resfile -s d *pdb --path /path/to/weblogo

This will output a sequence logo, as well as a .tab file that contains all amino acid frequencies at all positions. From this file you can convert amino acid frequencies into a bitscore (which is equal to $p_i \times \log_2(20 \times p_i)$) and calculate the native sequence recovery (defined as the bitscore of the native amino acid divided by total bitscore at a position).

Evolutionary Sequence Profiles

To generate an evolutionary sequence profile for each protein PSIBlast was run with the following command:

psiblast -query fyn.fasta -db non_redundant_database.db -num_iterations 2 -out out.txt -out_pssm fyn_pssm.txt -out_ascii_pssm fyn_ascii_pssm.txt

The ASCII PSSM contains amino acid frequencies for all positions in the FASTA file. I filtered by 1) residues that were specified in my resfile, and 2) residues that were mutated in the top ten models produced by any of the three design protocols. This evolutionary PSSM was then compared to the design PSSM for each design method. To do this I calculated a squared difference matrix between the two PSSMs, and summed the difference over all amino acids at a given position. At each position, I subtracted this value from 2 and normalized by a factor of 2 to yield a percent similarity score. I then averaged the percent similarity over all positions to generate an overall percent similarity score.
